# A Rare Case of Extra-Medullary Portacaval Plasmacytoma in a Patient With Relapsed Multiple Myeloma

**DOI:** 10.7759/cureus.26552

**Published:** 2022-07-04

**Authors:** Nyan A Bethel, Folasade Ajayi, Henna Asrar, Sahithi Chittamuri, Hamid Shaaban

**Affiliations:** 1 Internal Medicine, Saint Michael's Medical Center, Newark, USA; 2 Medical Education, St. Michael's Medical Center, Newark, USA; 3 Hematology and Oncology, Saint Michael's Medical Center, Newark, USA; 4 Hematology and Oncology, St. Michael's Medical Center, Newark, USA

**Keywords:** portacaval, plasma cells, radiation, extramedullary plasmacytoma, multiple myeloma

## Abstract

Multiple myeloma (Kahler disease) is a monoclonal plasma cell immunoproliferative neoplasm originating within the bone marrow that involves the production of monoclonal immunoglobulins, mostly IgG and IgA. Extramedullary plasmacytoma (EMP) is a subset of plasma cell neoplasms that can develop in patients at the time of diagnosis with multiple myeloma, or relapse of the disease. Symptoms related to plasmacytomas depend on the primary location. Here in, we present a rare case of extramedullary plasmacytoma involving the portacaval space in an 83-year-old African American female with relapsed multiple myeloma. She was treated successfully with radiation therapy with complete resolution of the mass. In this case report, we aim to discuss the clinical features along with diagnostic methods and treatment for extramedullary plasmacytomas with emphasis on utilizing a multidisciplinary approach in managing these rare cases.

## Introduction

Multiple Myeloma (MM) is a neoplastic proliferation of plasma cells inside the bone marrow. The infiltration of these cells into the bone marrow leads to the suppression of hematopoiesis [1]. This disorder accounts for approximately 10% of all hematologic malignancies. EMP is characterized by the neoplastic proliferation of plasma cells in soft tissues and accounts for 3% of all plasma cell neoplasms and of that percentage, 90% arise in the head and neck [2], while 4-5% specifically involve the GI tract, making it a rare entity. Patients with multiple myeloma can develop EMP at the time of diagnosis or at the time of relapse [3]. Irrespective of the time it is diagnosed, EMP remains a poor prognostic marker and a therapeutic dilemma [1]. In patients with already established MM, the diagnosis of EMP is made by radiologic modalities which include computed tomography, positron emission tomography (PET)/CT, magnetic resonance imaging (MRI), ultrasound, biopsy, or at times with physical examination [3, 4]. Treatment is dictated by the involved site of disease and can include radiation, and surgery with or without chemotherapy [5]. Here, we present a rare case of extramedullary plasmacytoma involving the portacaval space with a history of relapsed multiple myeloma. 

## Case presentation

An 83-year-old African American female with a past medical history of IgA kappa MM diagnosed in 2012, End-Stage Renal Disease (ESRD), non-insulin-dependent Type II Diabetes Mellitus, Hypertension, and Rheumatoid Arthritis presented to the emergency department (ED) with complaints of non-bloody, non-bilious vomitus and nausea. These episodes were associated with non-radiating generalized abdominal pain, unrelated to food consumption.

With regards to her history of Multiple Myeloma, she initially received 8 cycles of bortezomib, lenalidomide, and dexamethasone followed by 35 cycles of Lenalidomide and Dexamethasone for a duration of 8 months. Despite being on this regimen, her myeloma progressed to the point where she required hemodialysis. She was then started on Daratumumab and Dexamethasone for 11 months. A repeat skeletal survey showed the resolution of osteolytic lesions that were seen on the original scans at the time of diagnosis. Further workup showed continuing increase in IgA and serum-free light chain while on Daratumumab. Due to these findings, her regimen was switched to Elotuzumab, Pomalidomide, and Dexamethasone. 

**Figure 1:**
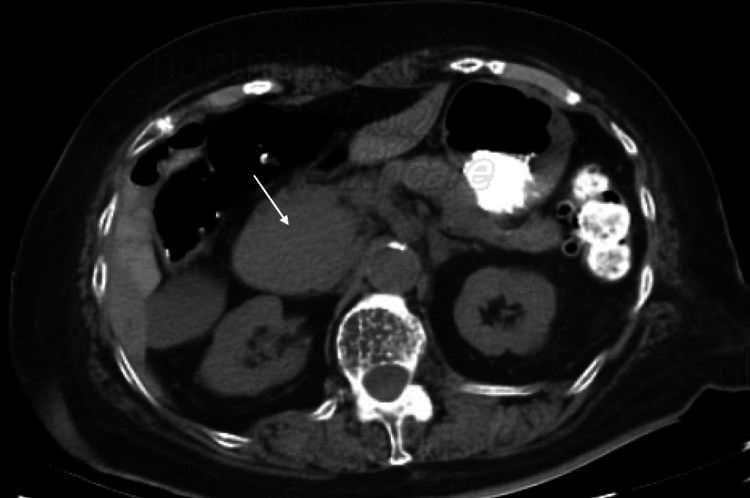
Axial computed tomography (CT) scan of the abdomen showing a portacaval mass (white arrow)

**Figure 2:**
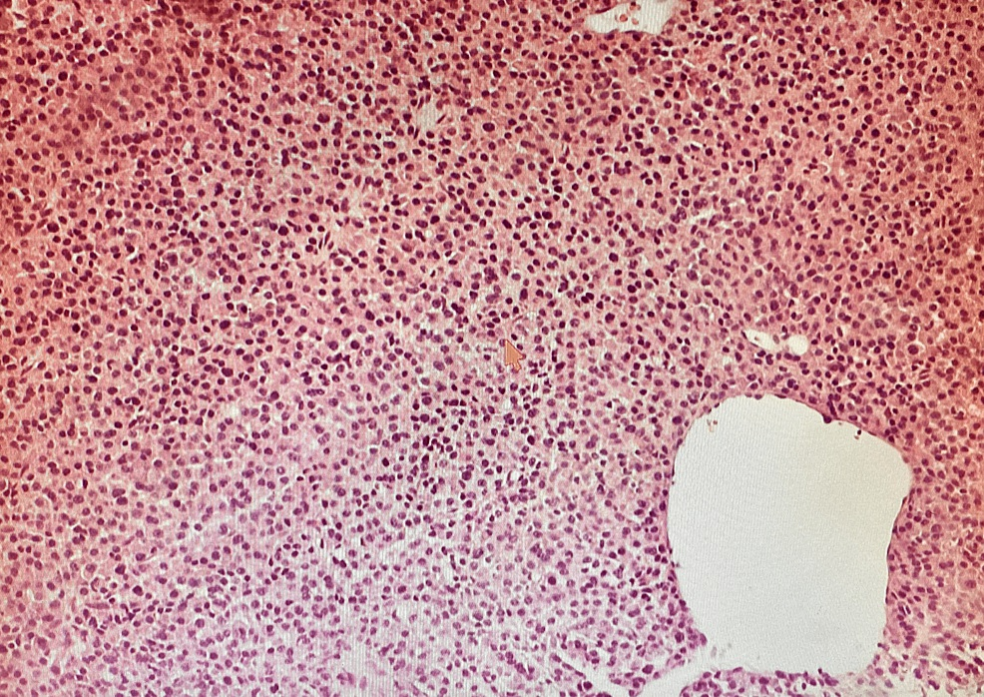
H&E stain of the mass showing infiltration of plasma cells (100x)

**Figure 3:**
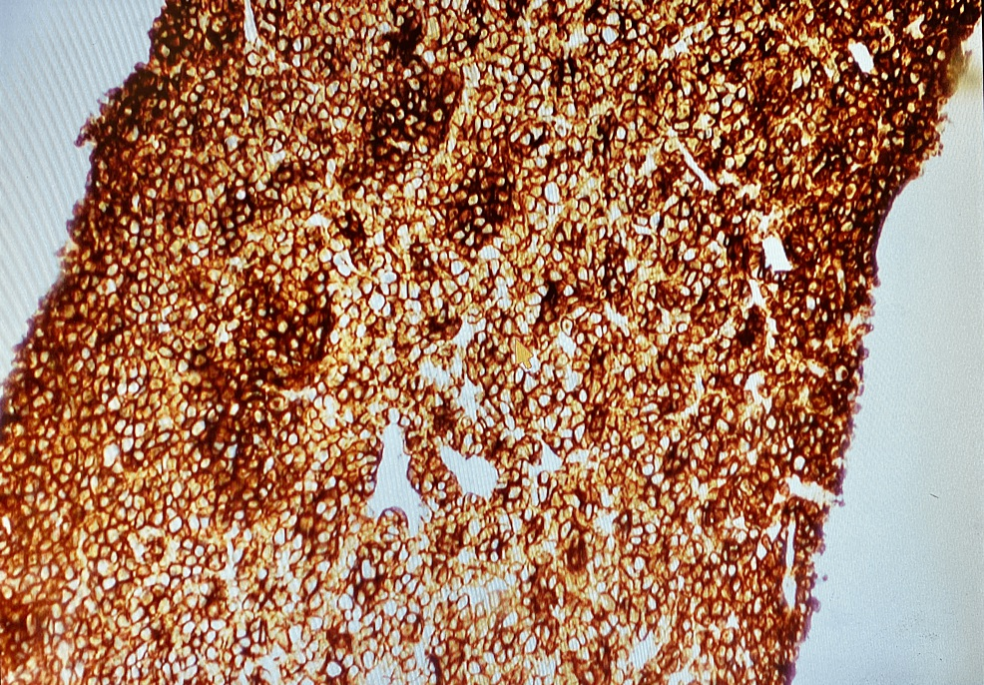
Immunohistochemical stain (CD138+) of the bile duct demonstrating plasma cells (100x)

**Figure 4:**
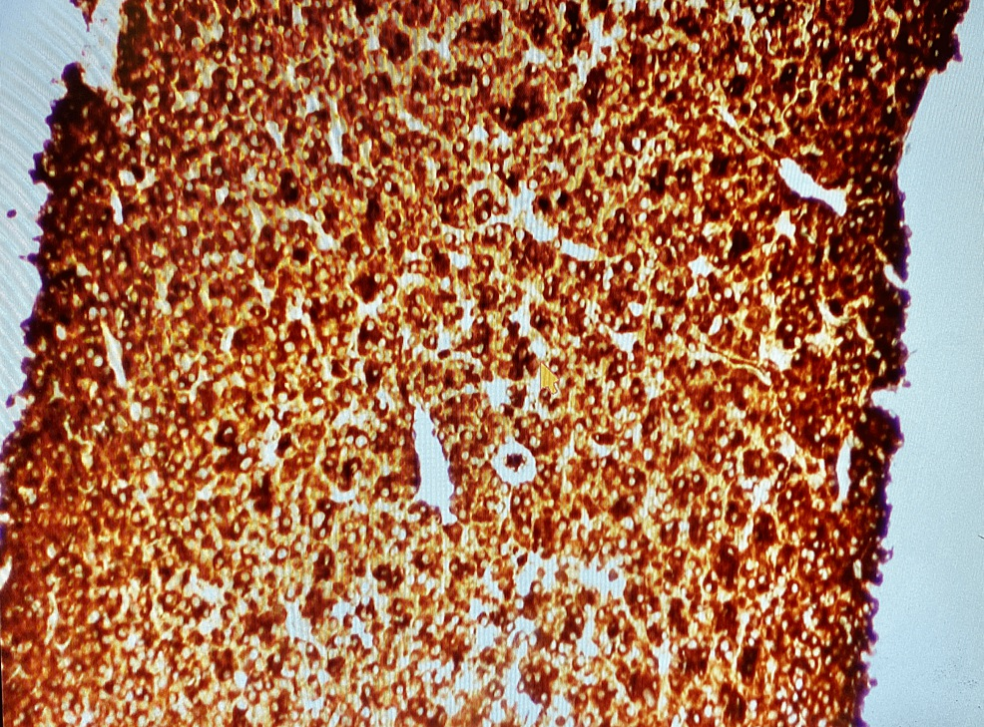
Immunochemical stain showing kappa predominance for myeloma (100x)

The patient presented approximately 9 years later from the time of diagnosis with the aforementioned symptoms. Vitals on admission were stable. On physical examination, the abdomen was non-distended with diffuse tenderness on deep palpation in all four quadrants, without guarding or rigidity. Bowel sounds were present throughout. Initial laboratory results showed hemoglobin of 8.5 with an HCT of 27.3 and MCV of 89.8 suggesting normocytic anemia (Table 1). Creatinine on admission was 1.6, which was at baseline. Albumin was 2.1 and serum calcium was 6.6, corrected calcium for albumin was 8.1 (Table 1). CT abdomen/pelvis with oral contrast showed a mass at the region of the pancreatic head and descending duodenum measuring 5x4x5.4 cm (Figure 1). Serum protein electrophoresis (SPEP) and urine protein electrophoresis (UPEP) showed only an isolated spike in beta-microglobulin. CEA and CA19-9 were obtained and were within the normal limits (Table 1). Hematology/oncology was consulted given the history of multiple myeloma. MRI of the abdomen without the use of contrast due to renal failure was obtained for further evaluation. The results showed that the mass was extrinsic to the pancreas and the duodenum, and likely represented an enlarged portacaval lymph node measuring 5.5cm. Invasive radiology was consulted and the patient underwent a CT-guided biopsy. H&E staining of the mass showed infiltration of plasma cells (Figure 2). Immunohistochemical staining identified CD138+ plasma cells (Figure 3) and kappa predominance (Figure 4). Ki67 staining labeled 30-40% of these cells. Flow cytometry demonstrated no discrete CD19^+^/CD20^+^ B cell population. Based on these findings, a diagnosis of extramedullary plasmacytoma of the portacaval space was made. 

**Table 1:** Key laboratory values

Test	Reference Value	Results
Hemoglobin	13.5-17.5 (g/dL)	8.5
Hematocrit	38-50 (%)	27.3
MCV	80-100 (f/L)	89.8
Platelets	150-450 (10*3µ/L)	278
BUN	6-24 (mg/dL)	32
Creatinine	0.6-1.2 (mg/dL)	1.6
Glucose	70-140 (mg/dL)	76
Alkaline Phosphatase	40-115 (U/L)	41
Total Protein	6.4-8.4 (g/dL)	5.3
Albumin	3.6-5.1 (g/dL)	2.1
Calcium	8.6-10.4 (mg/dL)	6.6
Lipase	73-393 (U/L)	46
CEA	0-3 (ng/ml)	0.3
CA19-9	0.34 (U/ml)	10.1

Radiation oncology was consulted and the patient completed 25 sessions of palliation radiation therapy at a dose of 45 Gy. Repeat CT imaging 4 months later showed complete resolution of the mass. The patient is currently being followed up for recurrence and/or development of plasmacytomas. 

## Discussion

Multiple Myeloma is a plasma cell disorder that accounts for approximately 10% of all hematologic malignancies [1]. It commonly affects more men than women at a 3:2 ratio and is twice as common amongst the African American population, with the average age of onset at 50-70 years [3]. According to the International Myeloma Working Group (IMWG), the diagnosis of multiple myeloma requires the presence of systemic manifestations including hypercalcemia, renal involvement, anemia, and lytic bone lesions (CRAB) and the presence of either one of the following: 10% clonal plasma cells on bone marrow biopsy or clonal bone marrow plasma cells >60%, serum-free light chain ratio >100, or more than one focal lesion on MRI [6,7]. 

On the continuum of plasma cell dyscrasias, solitary plasmacytoma is an entity that can be divided into SBP (intraosseous) or EMP (extraosseous) [8]. Narrowing in on EMP as it pertains to this case, as the name suggests, it occurs outside the bone marrow, often in soft tissues. In recent times, we have seen an increasing incidence of EMP in patients with MM [1]. This increased incidence can be attributed to the advancement in therapy to treat multiple myeloma, thus prolonging survival. By far, the most common site is the head and neck [5, 9]. Less common sites include the gastrointestinal tract at 4% followed by lungs, skin, and retroperitoneal organs [10]. Focusing on gastrointestinal tract involvement, an EMP can present with either mass effect or organ-specific dysfunction [4, 11]. This can mimic a GI malignancy and present with melena, hematochezia, jaundice, anemia, or intestinal obstruction leading to other symptomatic sequelae (nausea, vomiting, constipation, hydronephrosis). Other symptoms include but are not limited to abdominal pain, tenesmus, and perforated viscus in rare instances.

The incidence of EMP in newly diagnosed MM patients ranges between 7-18%. However, the mechanism of progression from MM to EMP is poorly understood. Some of the proposed and commonly accepted theories include a downregulation of adhesion molecules (VLA-4, CD44, and CD56) that normally function to anchor tumor cells to the endothelial basement membrane, chemokine receptors (CCR1, CCR2), and factors that play a critical role in basement membrane homing of myeloma cells (CXCR4 and its ligand, SDF-1alpha). Some research even suggests that increased angiogenesis is culpable for metastatic myeloma cells [12, 13]. In these ways, either through direct extension from the bone tumor or hematogenous spread, myeloma cells disseminate leading to EMP. Interestingly, Joan et al show that there is a higher incidence of EMP in MM relapsed patients who also receive allogeneic transplantation, especially in patients requiring dose-reduced conditioning therapy [13]. More studies need to be done to make a definitive conclusion using comparable groups of patients. 

Initial identification of an EMP is often accomplished by either axial imaging (CT, PET) or direct procedural visualization [3,14]. However, biopsy with immunohistochemistry of a resected specimen remains the gold standard [4]. In the gastrointestinal tract (GIT), endoscopic ultrasound fine-needle aspiration (FNA) has been commonly used for EMP diagnosis within the pancreas, liver, and gallbladder. As seen in our patient, CT imaging and biopsy with immunohistochemistry were utilized in arriving at the diagnosis. 

There is no gold standard that guides the management of GI plasmacytomas since its occurrence is quite rare. However, treatment often includes a combination of surgery with or without radiotherapy at a dose of 40-50 Gy over a 4-week period [2, 4, 15]. As demonstrated in our patient, she achieved complete resolution of the mass after completing 25 sessions of radiotherapy at a dose of 45 Gy. Li et al mentioned that in one case series there was an improved 5-year progression-free survival with radiotherapy alone versus a combination of radiotherapy and surgery [16]. However, this study did not exclusively study GI-originating plasmacytomas and failed to consider differences in tumor biology. Therefore, a multidisciplinary approach must be considered when managing these complex cases given the lack of data.

## Conclusions

Extramedullary plasmacytoma is rare and usually presents with either mass effect or organ-specific dysfunction. It is important to note that patients with the presence of extramedullary plasmacytoma after multiple myeloma relapses usually have a poor prognosis. Gold standard treatment for EMP remains unclear but suggests a combination of chemotherapy with radiation or even surgery. In patients presenting with relapses in multiple myeloma and the onset of any form of gastrointestinal symptoms which includes but is not limited to vomiting, diarrhea, hematochezia, melena, and constipation, oncologists should be aware to consider EMP involving the gastrointestinal system as part of the differential diagnosis, especially in patients with already established multiple myeloma. Since data is limited regarding the treatment of gastrointestinal tract EMP, a multidisciplinary approach must be taken by both oncologists and surgeons in managing these rare and complex cases.
